# Study on Viscoelastic Characteristics of Polymer Solution Formation and Their Effect on Oil Displacement Efficiency

**DOI:** 10.3390/polym18010002

**Published:** 2025-12-19

**Authors:** Shijie Zhu, Yong Zhu, Lijun Chen, Jie Zhang, Xueli Duan, Yunxiong Cai, Xinsheng Jiang

**Affiliations:** 1 College of Petroleum and Natural Gas Engineering, Chongqing University of Science and Technology, Chongqing 401331, China; zhusj@cqust.edu.cn (S.Z.);; 2 Joint Logistics Support Force Engineering University, Chongqing 401331, China; 3 Sinopec Zhongyuan Petrochemical Co., Ltd., Puyang 457000, China; 4 Binzhou Inspection and Testing Center, Dongying 257000, China

**Keywords:** polymer solution, heavy oil, viscoelasticity, resistance factor, residual resistance factor

## Abstract

Polymer solutions exhibit radial flow characteristics upon injection into a formation via the wellbore. Accurately characterizing their viscoelastic properties at varying seepage velocities and quantifying their impact on displacement efficiency are crucial for advancing polymer flooding technology. This study simulated shear rate variations during polymer injection and integrated laboratory-measured viscoelastic properties with permeability characteristics in porous media. An analysis of the oil displacement performance between viscoelastic polymer solutions and a purely viscous fluid, glycerol, was conducted. The key findings are as follows: (1) Polymer elasticity, characterized by the first normal stress difference, diminishes with decreasing injection time/solution concentration. Significant viscoelasticity is observed near the wellbore, weakening in deeper reservoir regions. (2) The polymer type and injection conditions govern the development of solution “effective viscosity” during porous medium flow. A fundamental trend under elevated flow velocities is an increase in effective viscosity with shear rate. (3) Comparison with glycerol demonstrates that the viscoelastic effect of polymer solutions enhances heavy-oil displacement efficiency. The magnitude of this viscoelastic effect within the porous medium directly correlates with its contribution to improved displacement efficiency.

## 1. Introduction

Polymer solutions employed for enhanced oil recovery are quintessential viscoelastic fluids [1]. These materials exhibit intermediate behavior between purely viscous Newtonian fluids and elastic solids, simultaneously manifesting both viscous dissipation and elastic energy storage. During porous medium flow below the yield stress, viscoelastic fluids experience viscous flow resistance; upon removal of shear stress, a portion of the deformation energy is recoverable due to elasticity. Research and development of polymer solutions focus on elucidating their characteristic properties and influencing factors, refining synthesis and formulation techniques, and ultimately enhancing their performance to meet specific oilfield development requirements [2]. The viscoelastic nature of these solutions fundamentally distinguishes their behavior from that of purely viscous fluids. Flow through porous media induces elastic deformation due to pore-throat constraints, generating an elastic viscosity component [3,4,5,6]. Consequently, the effective viscosity (*μ_eff_* = *μ_viscous_* + *μ_elastic_*) expressed during flow significantly exceeds that of equivalent viscous fluids. This enhanced effective viscosity translates to superior flow control capability, making viscoelastic polymers more effective for improving oil recovery [7,8,9,10,11,12]. Characterizing polymer viscoelasticity typically involves oscillatory rheometry, measuring storage modulus (G′) and loss modulus (G″) under small-amplitude oscillatory shear. However, data acquired at controlled oscillation frequencies often poorly represent the viscoelastic performance observed under actual reservoir flow conditions [13,14]. This discrepancy limits the practical applicability of laboratory-derived viscoelastic understanding. Bridging this gap between laboratory measurements and field performance, effectively translating fundamental viscoelastic insights to reservoir conditions, remains a significant research challenge. Accurately characterizing the viscoelastic behavior of polymer solutions during flow in porous media is thus a critical issue in polymer flooding technology. Establishing reliable characterization methods under porous medium conditions is essential to determine whether enhanced oil recovery stems from the viscoelastic properties themselves or merely from increased viscous forces. Two primary approaches exist: (1) Shear Rate Equivalence—This involves analyzing the linear velocity during injection to calculate an equivalent shear rate or oscillation frequency. Correlating this with laboratory rheological data provides an estimated viscoelastic response under reservoir conditions. While theoretically grounded, this method exhibits significant deviations from actual behavior but offers a preliminary assessment of whether a given polymer solution concentration exhibits viscoelasticity under target conditions. (2) Porous Medium Flow Experiments—This entails directly measuring flow properties, e.g., resistance factor and residual resistance factor, in core samples. Analysis yields an effective viscosity reflecting the combined viscous and elastic effects, thereby demonstrating the viscoelastic characteristics within porous media [15,16,17].

Therefore, utilizing a polymer-flooded reservoir in an offshore oilfield as a case study, the following experimental sequence was implemented: (1) Comprehensive rheological characteristics, including viscosity modulus and energy dissipation modulus, of polymer solutions at varying concentrations were initially evaluated [18]. (2) The viscoelastic behavior of polymer solutions under reservoir conditions was subsequently analyzed by employing equivalent conversion between shear rate and linear velocity. (3) Resistance coefficient and residual resistance coefficient measurements were then conducted to validate the manifestation of viscoelasticity within porous media. (4) Conclusively, comparative core flooding experiments employing viscoelastic polymer solutions and viscous glycerol solution were performed on heavy-oil systems. This elucidated the viscoelastic effect on heavy-oil displacement dynamics, thereby providing foundational insights to underpin the advancement of polymer flooding technology for heavy-oil reservoirs.

## 2. Viscoelastic Characteristics of Polymers Under Reservoir Conditions

This section employs numerical simulation to analyze shear rate variations in porous medium flow. By integrating laboratory-derived viscoelastic properties at different shear rates, we systematically characterize the viscoelastic behavior of polymers across reservoir locations and injection concentrations.

### 2.1. Characteristics of Changes in Polymer Solution Concentration and Shear Rate Under Injection Conditions

**Figure 1:**
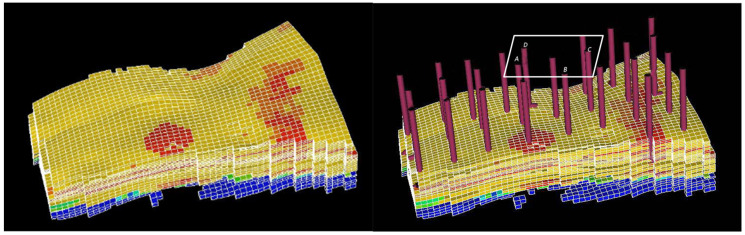
Geological model of the target block.

The viscoelastic properties of the SNF3640C polymer solution under reservoir conditions constitute the prerequisite for conducting displacement experiments. Accordingly, a geological reservoir model was developed using Tinavigator 20 numerical simulation software, reflecting the target reservoir’s characteristics, shown in Figure 1.

The simulation parameters utilized are shown in Table 1 [18,19].

The expression of viscoelasticity in polymer solutions is mainly influenced by two aspects. One is the shear rate during the polymer injection process, and the other is the effective solution concentration after diffusion, dilution, adsorption, and retention of the polymer solution after injection into the formation. The linear velocity characteristics and effective solution concentration changes during the injection of polymer solution with a concentration of 1750 mg/L into the reservoir were studied using mathematical models. The variation in linear velocity during polymer solution injection was analyzed based on reservoir numerical simulation, and the shear rate was converted using Formula (1) [20]. The results are shown in Table 2.

(1) $$\gamma =\frac{3n+1}{4n}\frac{v}{{\left(\frac{k}{2\phi }\right)}^{\frac{1}{2}}}$$

In the formula, *n* is obtained based on the power law coefficient of the target polymer under different concentration conditions; *K* is permeability, mD; *v* is linear velocity, m/s; and φ is porosity, %.

**Figure 2:**
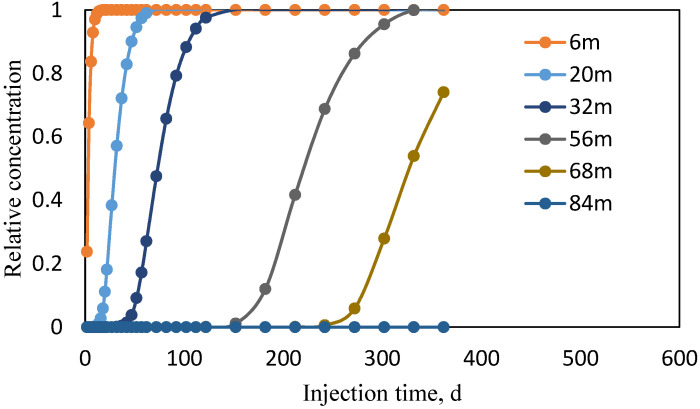
Relative concentration changes in polymer solution at different positions and times.

Reservoir simulation results, shown in Table 2, indicate that polymer solutions experience predominantly low shear rates < 0.6 s^−1^ following injection, with elevated shear rates confined to the near-wellbore region and perforations. Beyond the near-wellbore zone, shear rates throughout the reservoir remain within this lower range. This distribution demonstrates that polymer solutions primarily encounter low-shear conditions under reservoir-scale flow. Further analysis of polymer propagation behavior was conducted through reservoir simulation, as shown in Figure 2, revealing the spatial evolution of solution concentration after injection.

Following injection, polymer solution concentration exhibits spatial attenuation due to adsorption/retention and dispersion effects, resulting in progressive dilution with propagation distance. Propagation velocity decreases radially from the wellbore, yielding shorter concentration stabilization times near the injection point and extended equilibration periods in distal regions. Consequently, the leading edge maintains prolonged low-concentration conditions, where viscoelastic properties become strongly concentration-dependent.

**Figure 3:**
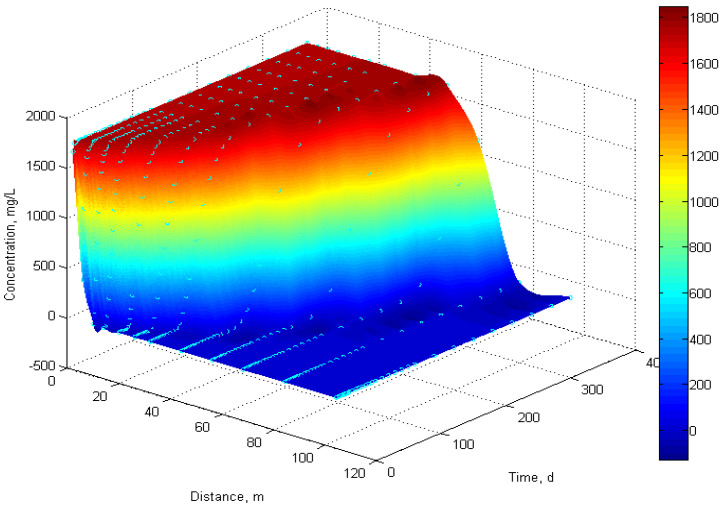
Concentration changes during polymer injection process.

Spatial analysis of velocity and concentration fields via MATLAB 2010a, shown in Figure 3, reveals distinct flow regimes: (1) near-wellbore zone—elevated concentrations > 1750 mg/L and shear rates; (2) far-field regions—diluted concentrations < 500 mg/L and low shear rates < 0.6 s^−1^.

### 2.2. Viscoelastic Characteristics of Polymer Solutions

**Figure 4:**
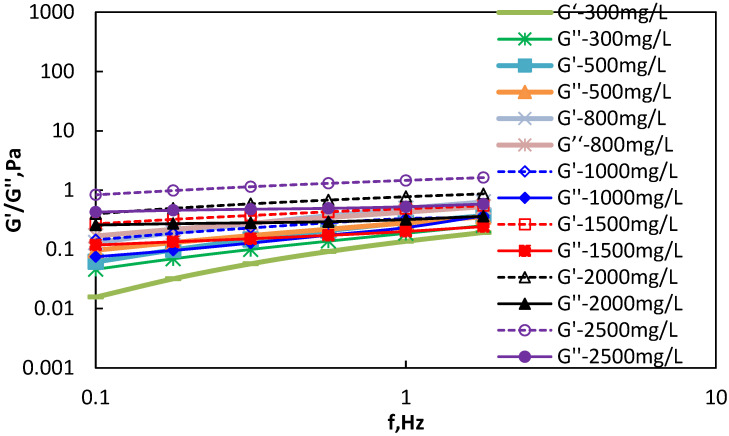
Viscoelasticity of polymer SNF3640C.

The hydrophobically associating polymer SNF3640C (Aisen Flocculant Co., Ltd., Jiangsu, Taizhou, China) was selected for rheological characterization [21,22]. Solutions were prepared at concentrations of 300, 500, 800, 1000, 1400, 2000, and 2500 mg/L. Oscillatory rheometry was performed using an RS600 rheometer with frequency sweeps from 0.1 to 1.77 Hz. The resulting loss modulus (G″) and storage modulus (G′) measurements for SNF3640C solutions are presented in Figure 4.

For the hydrophobically associating polymer SNF3640C, viscoelastic behavior below the critical concentration (C) exhibits crossover points between loss modulus (G″) and storage modulus (G′). At concentrations exceeding C, all three tested solutions demonstrate consistently greater storage modulus (G′) than loss modulus (G″). This indicates that within the studied oscillatory frequency range, SNF3640C’s viscoelastic response is primarily elastic-dominated. At low concentrations, SNF3640C maintains elastic dominance, with its strong intermolecular associations significantly enhancing resistance to deformation.

### 2.3. Characterization of Viscoelasticity of Polymer Solutions at Different Locations and Injection Concentrations

The first normal stress difference (N1) constitutes the primary metric for characterizing elastic behavior in viscoelastic fluids [21,22]. In porous medium flows, N1 quantitatively describes polymer solution elasticity. Complementary viscoelastic metrics can be derived by correlating shear-dependent viscosity profiles with shear rate and linear velocity relationships. The viscoelastic properties of polymer solutions were evaluated through comparative analysis using the empirical N1 calculation formula for polymers, Formula (2).

(2) $$N_{1}=2G^{\prime }{\left[1+{\left(\frac{G^{\prime }}{G^{\prime \prime }}\right)}^{2}\right]}^{0.7}$$

**Figure 5:**
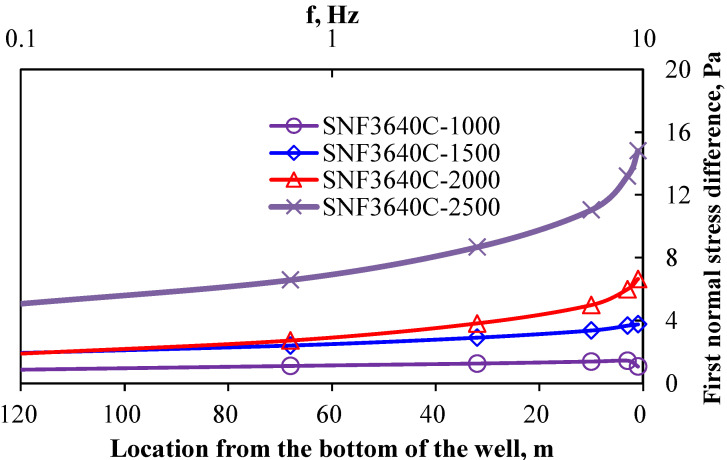
First normal stress difference of polymer SNF3640C.

The first normal stress variation relationship after injecting polymer solution into the formation is shown in Figure 5.

**Figure 6:**
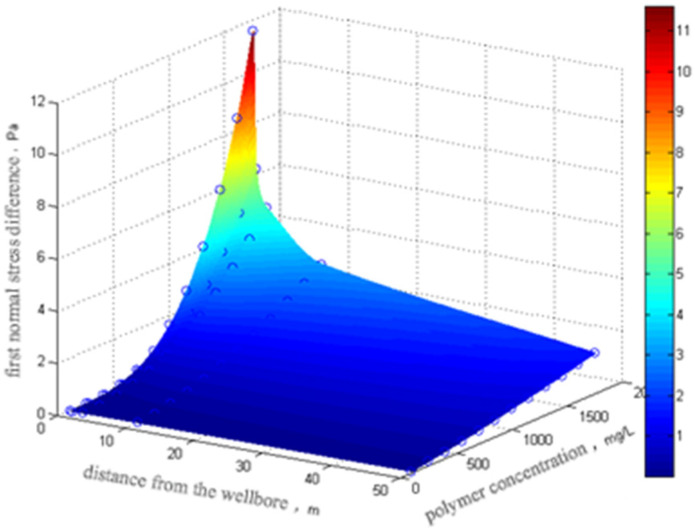
First normal stress difference after polymer injection.

Analysis of the first normal stress difference (N_1_) for polymer SNF3640C (produced by Aisen Company in France, Product Code) indicates the following: At low concentrations, 1000 and 1500 mg/L, N_1_ exhibits limited enhancement with increasing oscillation frequency. Under high-concentration conditions, 2000 and 2500 mg/L, N_1_ demonstrates substantial amplification, conferring pronounced elastic characteristics. Near the wellbore, SNF3640C maintains significant N_1_ values despite continuous polymer concentration fluctuations during injection/production cycles. Based on the established concentration profile of the injected polymer system’s previous sequence, N_1_ was analyzed as a function of both concentration and radial distance from the wellbore, as shown in Figure 6 [22,23].

As depicted in Figure 6, increasing injection duration correlates with progressive elevation of polymer concentration and concomitant enhancement of the first normal stress difference (*N*_1_). This trend signifies intensified viscoelastic effects within the porous medium. With increasing propagation distance, the superficial flow velocity diminishes, resulting in gradual reduction in *N*_1_. Consequently, the primary function of the polymer system in subsurface formations remains viscoelastic flow control in high-concentration, high-shear-rate regions—specifically the near-wellbore zone. Figure 6 further demonstrates that despite propagation to 50 m reservoir depth, the polymer maintains sufficient concentration to exhibit discernible viscoelastic response.

## 3. Viscoelastic Characteristics of Polymers Under Infiltration Experiments

To investigate viscoelastic flow behavior in porous media, SNF3640C—a polymer exhibiting enhanced viscoelastic properties—was selected as the representative viscoelastic fluid. The solution concentration was 1500 mg/L, yielding an apparent viscosity of 64 mPa·s. For comparative analysis, 70% glycerol, with an apparent viscosity of 65 mPa⋅s, served as the viscous Newtonian control fluid. This experimental design maintains near-identical apparent viscosities across displacement systems, enabling isolation of elasticity effects during oil displacement efficiency studies.

### 3.1. Pressure Characteristics of Viscoelastic Polymers in Porous Media

Permeation characteristics of polymer solutions in porous media were evaluated using conventional experimental methods [24,25]. Injection pressure profiles were quantified at flow rates of 0.1, 1, and 10 mL/min. The classical Darcy’s law was employed to calculate displacement pressures for a pure viscous glycerol system under identical injection conditions. Comparative analysis of pressure differentials between the glycerol control and polymer systems enables assessment of elastic flow resistance development across injection velocities. Results are presented in Table 3.

Table 3 demonstrates that 65 mPa⋅s glycerol injected at 0.1 mL/min yields an injection pressure of 0.0087 MPa when calculated via Darcy’s law for the target core medium. Under identical conditions, the polymer system exhibits significantly elevated injection pressure relative to the glycerol system. This differential confirms viscoelastic response during polymer permeation through porous media, establishing greater flow resistance than purely viscous fluids. The resultant pressure differential manifests this phenomenon. During viscoelastic fluid transport in porous media, pore-throat geometries induce extensional deformation—distinct from viscous fluid behavior. This generates elastic stresses and enhanced flow resistance, thereby enhancing displacement-front control.

### 3.2. Seepage Characteristics of Polymer Solutions Under Variable Velocity Conditions

Permeation behavior of viscoelastic polymer solutions was characterized across incremental flow rates using the following experimental protocol [26,27]: (1) Polymer solution was injected into crude oil-saturated cores (K = 2000 mD) at an initial rate of 0.1 mL/min until pressure stabilization. (2) Flow rates were sequentially increased to 0.2, 0.5, 1, 2, 5, and 10 mL/min following pressure equilibration at each preceding rate. (3) Injection pressures were recorded throughout all stages. Subsequent water flooding commenced at 0.1 mL/min, with stepwise rate increases at 0.2–10 mL/min implemented after each pressure stabilization. Corresponding injection pressures were documented. Resistance and residual resistance coefficients, RF and RRF, were calculated from pressure data using Equations (3) and (4). Effective viscosity (*μ_eff_* = *μ_viscous_* + *μ_elastic_*) was then determined via Formula (5), serving as the metric for quantifying polymer viscoelasticity within porous media [28,29].

*RF* = *P_p_*/*P_w_* (3)

*RRF* = *K_wb_*/*K_wa_* (4)

*μ* = *RF*/*RRF* (5)

**Figure 7:**
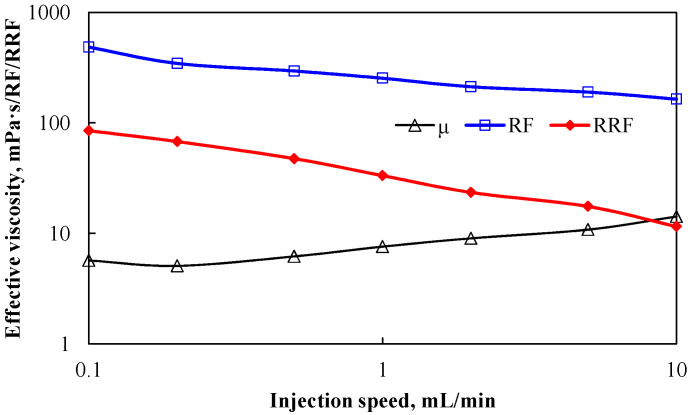
Effective viscosity of polymer solution in porous media.

Based on the experimental data and the summary calculation of Formulas (3)–(5), the experimental results in Figure 7 were obtained, which shows the effective viscosity of the polymer solution in porous media.

As injection rate increases, both *RF* and *RRF* for polymer solutions in porous media demonstrate decreasing trends. Analysis indicates viscosity-dominated flow behavior at low rates, where shear thinning reduces *RF*. The *RRF* decline is attributed to attenuated polymer adsorption retention at elevated velocities. The calculated effective viscosity (*μeff*) exhibits a gradual increase, primarily resulting from RF’s slower decline relative to *RRF*. Alternatively, this progression is attributable to enhanced elastic contributions (*μ_elastic_*) at higher injection rates, driving *μeff* elevation. This methodology resolves experimental challenges in quantifying effective viscosity and provides a quantitative metric for polymer permeation characteristics. Results further confirm that polymer flow behavior in porous media is governed by interdependent factors—including media properties, injection parameters, and solution rheology—requiring comprehensive consideration when evaluating viscoelastic effects under specified conditions.

## 4. Study on Influence of Viscoelasticity on Oil Displacement Effect

### 4.1. Oil Displacement Experiment Design

To mitigate diffusion and dilution artifacts during glycerol displacement, cores measuring 25 × 800 mm were selected, with average porosity of 29–32 [30,31,32]. The displacement protocol excluded initial water flooding to prevent leading-edge dilution and omitted post-displacement water flooding to avoid concentration degradation behind the displacement front. Displacement agent was continuously injected into oil-saturated cores until effluent production ceased (the crude oil has a viscosity of 70 mPa·s after degassing and dehydration under ground conditions; by saturating crude oil with variable flow rates, saturation experiments were completed, specifically by saturating it with 0.1 mL/min, 0.2 mL/min, 0.5 mL/min, 1 mL/min, 2 mL/min, and 5 mL/min for 4 h, 2 h, 1 h, 0.5 h, 0.2 h, and 0.1 h, respectively). Based on established effective viscosity characteristics of polymer solutions in porous media, five injection rates were selected to simulate reservoir positions: wellbore-proximal region (5 mL/min); near-wellbore zone (1 mL/min); 2 m radial distance (0.5 mL/min); 10 m radial distance (0.1 mL/min); and 100 m radial distance (0.01 mL/min). The experimental core data statistics are shown in Table 4.

### 4.2. The Influence of Viscoelasticity on Oil Displacement Effect

**Figure 8:**
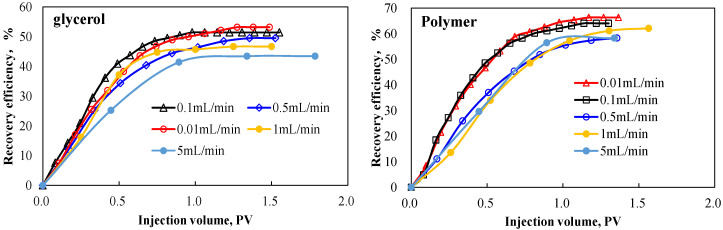
Oil displacement effect of glycerol and polymer.

Comparison of oil displacement efficiency between glycerol and polymer solutions at different displacement rates is shown in Figure 8 and Table 5.

Polymer SNF3640C demonstrates significantly higher oil displacement efficiency than the glycerol system at elevated injection rates, with minimal efficiency disparity observed at 0.5 mL/min. Displacement efficiency for glycerol progressively decreases with increasing injection rate, exhibiting the most pronounced decline at higher velocities. The polymer system attained peak efficiency 62.14 at 1 mL/min, surpassed only by performance at 0.1 mL/min and 0.01 mL/min. This demonstrates that injection rate critically governs the activation and manifestation of polymer viscoelasticity in porous media, confirming that viscoelastic properties significantly enhance heavy-oil recovery efficiency.

## 5. Conclusions

(1) Following the injection of polymer solution into the reservoir, the solution encounters varying shear rates during radial flow, leading to distinct viscoelastic characteristics at different spatial positions. As propagation continues outward, the effective concentration of the solution progressively diminishes, resulting in a corresponding reduction in its elastic properties. Consequently, polymer solutions demonstrate superior viscoelastic characteristics within the near-wellbore region, whereas their capacity to provide the strong viscoelasticity essential for effective flow control in deeper reservoir regions diminishes significantly.

(2) The viscosity evolution of polymer solutions during seepage through porous media deviates from conventional shear-thinning rheological behavior observed in laboratory settings. The solution’s viscosity is influenced by its elastic properties, with the fundamental trend under elevated flow velocities being an increase in effective viscosity with increasing shear rate.

(3) Viscoelastic polymer solutions exhibit enhanced flow control capabilities within porous media through elastic effects, which further improves heavy-oil recovery efficiency compared to viscosity control alone. Moreover, the efficacy of the viscoelastic effect in porous media is directly proportional to its contribution to enhanced oil recovery efficiency.

## Figures and Tables

**Table 1:** Simulation parameters utilized.

Type	Parameters
Injection Schemes	1. Waterflood baseline: 0.06 PV·a^−1^ injection rate. 2. Polymer flood: 0.3 PV slug size at 1750 mg/L concentration, 0.06 PV·a^−1^ injection rate
Reservoir Properties	1. Thickness: 200–400 m. 2. Initial pressure: 14.2 MPa. 3. Permeability: 2500 mD, coefficient of variation *V_K_* = 0.6. 4. Porosity: 32% average. 5. Initial oil saturation: 56%.
Fluid Properties	1. Crude oil viscosity: 65 mPa·s. 2. Oil formation volume factor: 1.11. 3. Injection water salinity: 9374 mg/L. 4. Temperature: 65 °C.

**Table 2:** Linear velocities and corresponding shear rates at different positions from the bottom of the well.

Segmentation	Perforated Interval		Near-Well Zone				Exceeding 6 m			
Distance from the bottom of the well	0.01 m	0.3 m	0.5 m	0.8 m	1 m	2 m	6 m	20 m	56 m	104 m
Linear velocity	318.471	10.616	6.369	3.981	3.185	2.588	1.127	0.758	0.377	0.242
shear rate	171.502	5.717	3.430	2.144	1.715	1.394	0.607	0.408	0.203	0.13

**Table 3:** Stable pressure of the system at different injection rates.

Differential Pressure, MPa	Injection Rate, mL/min		
	0.1	1	10
Glycerol	0.0087	0.087	0.87
SNF3640C	0.4	2.16	11.5

**Table 4:** Analysis of the oil displacement efficiency of polymer/glycerol at different speeds.

Core Number	Pore Volume	Saturated Oil Quantity	Displacing Velocity, mL/min	Porosity	Oil Saturation	Displacement Fluid System
2#	12.246	10.5 mL	0.1	32.20%	0.86	glycerol
13#	11.8	9.9 mL	0.5	30.00%	0.84	glycerol
15#	11.3	9.4 mL	0.01	28.80%	0.83	glycerol
21#	12	10.1 mL	1	32.20%	0.84	glycerol
22#	11.2	9.4 mL	5	28.50%	0.84	glycerol
14#	12.2	10.8 mL	0.5	31.08%	0.89	SNF3640C
16#	12.3	10.7 mL	0.01	31.30%	0.87	SNF3640C
27#	11.2	9 mL	5	31.08%	0.8	SNF3640C
25#	11.5	9.8 mL	1	31.30%	0.85	SNF3640C
16#	12.3	10.7 mL	0.01	31.30%	0.87	SNF3640C

**Table 5:** Difference in displacement efficiency between glycerol and polymer.

Displacing Velocity, mL/min	Oil Displacement Efficiency of Glycerol	Oil Displacement Efficiency of Polymer	Difference
0.01	53.19%	66.36%	13.17%
0.1	51.43%	64.08%	12.65%
0.5	49.49%	58.33%	8.84%
1	46.67%	62.14%	15.47%
5	43.43%	58.33%	14.90%

## Data Availability

The original contributions presented in this study are included in the article. Further inquiries can be directed to the corresponding author.
